# Adaptation of Land-Use Demands to the Impact of Climate Change on the Hydrological Processes of an Urbanized Watershed

**DOI:** 10.3390/ijerph9114083

**Published:** 2012-11-12

**Authors:** Yu-Pin Lin, Nien-Ming Hong, Li-Chi Chiang, Yen-Lan Liu, Hone-Jay Chu

**Affiliations:** 1 Department of Bioenvironmental Systems Engineering, National Taiwan University, No. 1, Section 4, Roosevelt Road, Da-an District, Taipei City 106, Taiwan; Email: yplin@ntu.edu.tw (Y.-P.L); lchiang@ntu.edu.com.tw (L.-C.C); clickvito@gmail.com (Y.-L.L); 2 Environment and Energy Management Research Center, Overseas Chinese University, No. 100, Chiao Kwang Road, Taichung, 407, Taiwan; 3 Department of Geomatics, National Cheng Kung University, No.1, University Road, Tainan City 701, Taiwan; Email: honejay@mail.ncku.edu.tw

**Keywords:** adaptation, hydrological components, climate change, land use management

## Abstract

The adaptation of land-use patterns is an essential aspect of minimizing the inevitable impact of climate change at regional and local scales; for example, adapting watershed land-use patterns to mitigate the impact of climate change on a region’s hydrology. The objective of this study is to simulate and assess a region’s ability to adapt to hydrological changes by modifying land-use patterns in the Wu-Du watershed in northern Taiwan. A hydrological GWLF (Generalized Watershed Loading Functions) model is used to simulate three hydrological components, namely, runoff, groundwater and streamflow, based on various land-use scenarios under six global climate models. The land-use allocations are simulated by the CLUE-s model for the various development scenarios. The simulation results show that runoff and streamflow are strongly related to the precipitation levels predicted by different global climate models for the wet and dry seasons, but groundwater cycles are more related to land-use. The effects of climate change on groundwater and runoff can be mitigated by modifying current land-use patterns; and slowing the rate of urbanization would also reduce the impact of climate change on hydrological components. Thus, land-use adaptation on a local/regional scale provides an alternative way to reduce the impacts of global climate change on local hydrology.

## 1. Introduction

The conversion of land-use to provide food and shelter in response to increase human activity is one of the major modes of human modification of the global environment [1], as well as changes in the local environment, such as the hydrological processes at the watershed scale [2]. Meanwhile, the impact of global climate change is mediated at regional and local scales by biophysical processes associated with land-use and land-cover (LULC) [3]. For instance, changes in the global climate have a significant impact on local and regional hydrological regimes and processes, which in turn affect ecological, social and economical systems [4]. It is clear that both climate change and land-use change are important drivers of changes in a watershed’s hydrology; however, their relative effects are difficult to separate empirically [5], especially in watershed land-use planning and management. Moreover, the effects of climate change on land-use should be considered from two perspectives: (1) how land-use might be altered by climate change; and (2) what land management strategies would mitigate the negative effects of climate change [6]. Therefore, adapting land-use patterns is an essential aspect of strategies designed to minimize the negative outcomes of the now-unavoidable climate change at regional and local scales, including adapting watershed land-use patterns to accommodate the impact of climate change on a region’s hydrology. 

Adaptive capacity is defined as the “potential, capability, or ability of a system to adapt to climate change stimuli [7]. This implies that, theoretically, adaptive capacity is a system’s potential to reduce the damage caused by climate change, or to exploit its benefits. The impacts of climate change on environmental systems, such as hydrological processes, are gradually and cumulatively spreading from the global scale to local scales. Actions associated with building adaptive capacity may include communicating information about climate change, building awareness of the potential impacts of such change, maintaining the well-being of residents, protecting property/land, maintaining economic growth, and exploiting new opportunities [8]. Increasing the ability of environmental systems to adapt, or strengthening their adaptive capacity, is already an important consideration in responding to climatic changes [9]. Therefore, adaptation strategies and decisions are more likely to focus on reducing the cumulative impacts of climate change, and ensuring that the distributional impacts of adaptation are minimized [8]. In national, regional and local land-use planning, the impact of global climate change is mediated at regional and local scales by biophysical processes associated with land-use and land-cover (LULC) [3], such as the hydrological processes associated with land-use and land-cover. For example, the impact of climate change on water availability and quality will probably threaten the sustainability of water uses and increase the risk of lacking water for social and ecological systems [9]. Moreover, land-use is a key factor that must be considered when predicting potential future hydrological responses of a watershed [10], and then can be adapted to minimize the impacts of climate change on hydrological processes. Assessing the effects of land-use on a region’s hydrology is of special interest when discussing the expected effects of climate change [10]. Some recent studies assessed how land-use patterns and climate change singly and jointly affect a region’s hydrology [5,10,11,12,13,14] (e.g., The studies found that the combination of land-use patterns and climate change can result in more significant hydrological changes than either driver acting alone. Therefore, ways to increase the adaptive capacity of environmental systems, including incorporating the impact of climate change into development planning programs, become one of essential works for adapting climate changes.

The importance of LULC activities lies in the fact they represent important adaptation strategies. The studies [15,16,17] observed that, since climate change is a multi-dimensional issue, LULC must be included in global and regional strategies to mitigate the effects of climate change. The study [3] also suggested that LULC patterns can be used as biophysical tools to offset specific adverse aspects of climate change at local and regional scales. More specifically, they posited that new research could turn biophysical mechanisms into practical adaptation strategies rooted in the management of land-use and land cover patterns and processes. Because LULC-based strategies do not depend on remote political processes, they can be exploited at local and regional levels to help achieve local/regional conservation goals Pyke and Andelman argue that this empowers land managers to consider how their actions may contribute to adaptation, or may be maladaptive, under future conditions. Hence, land-use strategies will probably continue to evolve over the next few decades to adapt to climate change as well as in response to global and regional economic changes. These factors will have an important influence on the hydrology of watershed regions [10,18].

The objectives of this study are to: (1) simulate variations in the range of hydrological components induced by climate change; (2) determine land-use demand scenarios based on the hydrological simulations; (3) allocate land-use patterns based on the various land-use demands; and (4) compare the adaptive capacity of land-use demands to impacts of climate change on the hydrological components. We utilize three computer models in the study: a downscaling model to obtain local climate conditions from six General Circulation Models (GCMs), a hydrological model to simulate hydrological components, and a land-use change model to allocate land-use activities in the Wu-Tu watershed in northern Taiwan.

## 2. Materials and Methods

### 2.1. Study Area

**Figure 1:**
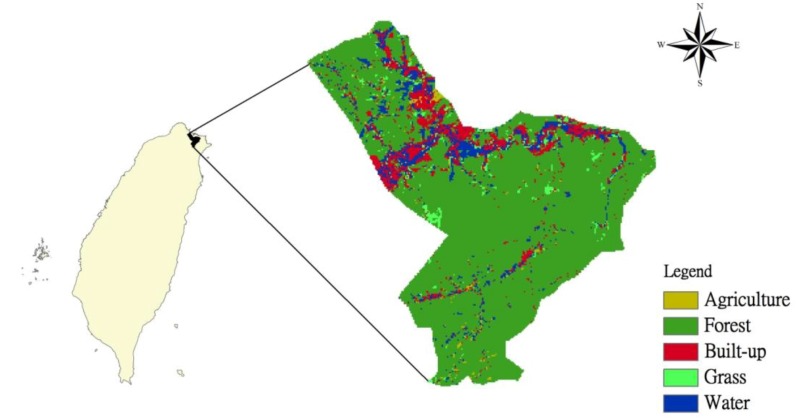
Location and land use types of the Wu-Tu Watershed.

The Wu-Tu watershed, which is located to the north of Taipei (Figure 1), is a sub-watershed of the Keelung Basin. It covers an area of 204.41 km^2^; and the average elevation and slope are 242 m and 0.005, respectively. The population of the watershed has grown rapidly in the last twenty-five years due to the expansion of the Taipei metropolitan area. Wu-Tu is actually a satellite city of Taipei. The watershed became urbanized between 1987 and 1997, with a population increase of 2.7% per year during that period. Land use patterns changed rapidly as a consequence, and the built-up area located in the downstream part of the watershed grew in line with the population. However, since 1997, the average annual population growth rate has slowed to approximately 1.05% [19].

### 2.2. Climate Change Scenarios

In the simulations, we used temperature and precipitation data from the following six General Circulation Models (GCMs): GFDL21 (Geophysical Fluid Dynamics Laboratory, NOAA, CM2.1), EHAM5 (Max Planck Institute for Meteorology, Germany, ECHAM5/MPI-OM), CGCM2 (Meteorological Research Institute, Japan Meteorological Agency, MRI-CGCM2.3.2), CCSM (National Center for Atmospheric Research, NCAR, Community Climate System Model, version 3.0), INCM3 (Institute of Numerical Mathematics, Russian Academy of Sciences, INMCM3.0), and HADCM3 (Hadley Centre for Climate Prediction and Research, Met Office, United Kingdom, HadCM3). All the data for the GCMs were obtained from the Data Distribution Centre of the Intergovernmental Panel on Climate Change (IPCC). The baseline scenario is defined as the weather condition during 1980–1999, and the A2 (medium–high) greenhouse gas (GHG) emissions scenario was selected as future climate scenario during 2010–2039 in this study. Since the spatial resolutions of GCMs are too coarse to represent local climate characteristics in Taiwan, the technique of simple downscaling between the baseline and the climate scenario of the nearest GCM grid was applied directly. The changes in temperature and precipitation as well as historical data were then used to generate weather data. We utilized the weather generation model [20] to generate daily temperature and precipitation data for the target climate scenarios. Daily precipitation data were derived by analyzing the random distribution of precipitation associated with an exponential distribution. In total, three hundred years of daily weather data were generated for the baseline and climate scenarios so that we could produce as many combinations of weather variability as possible.

It is assumed that changes in future precipitation in the study area will be the same as the difference between the future precipitation simulated by the GCMs and the current conditions at the nearest grid point [20]. Then, future climate scenarios can be estimated as follows. The change in precipitation is the ratio of the future precipitation rate to the current rate. It is calculated as follows[20]:

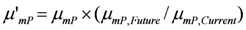
(1)
where *μ'_mP_*, *μ_mP_* and *μ'_mP_* are the current and future mean monthly precipitation rates (cm) respectively; and *μ_mP,Current_* and *μ_mP,Future_* are the simulated mean monthly precipitation rates (cm) under the current (the annual average for 1980–1999) and future (the annual average for 2010–2039) climate conditions, respectively. 

### 2.3. Adaptation of Land-Use Demands

Table 1 shows the land use patterns for various cases of land-use adaptation. Case 0 shows land-use change under the current land-use policy; and Cases 1–7 show adaptations of land-use patterns to mitigate the impacts of climate change. The land-use patterns in Cases 1~5 are proportional to those of Case 0 from 1999 to 2020. It is assumed that land use change linearly by time. For example, in Case 0, the original built-up area was 1187 ha in 1999, but the predicted built-up area in 2020 will be 1,623 ha (8.8%). Based on the adaptation policies in Cases 1~5, the built-up areas in 2020 will cover 1,536, 1,449, 1,362, 1,274, and 1,187 ha respectively. In other words, the proportion of built-up areas will decline to 8.3%, 7.9%, 7.4%, 6.9% and 6.5% of the watershed. In Case 6, the grassland will be converted to forest, and other areas will be same as Case 5. Moreover, in Case 7, the grassland and agricultural land will be converted to forest when compared to Case 5.

**Table 1:** Land use adaption scenarios (unit: ha).

Scenarios		Built-up	Forest	Agriculture	Grass	Water
Case 0	Demand	1,623	14,833	177	473	1,293
Case 1	Demand	1,536	14,898	186	486	1,293
	Adapted area	−87	65	9	13	0
		(−0.47%)	-0.35%	-0.05%	-0.07%	0.00%
Case 2	Demand	1,449	14,964	195	499	1,293
	Adapted area	−174	131	18	26	0
		(−0.95%)	-0.71%	-0.10%	-0.14%	0.00%
Case 3	Demand	1,362	15,029	204	512	1,293
	Adapted area	−261	196	27	39	0
		(−1.42%)	-1.07%	-0.15%	-0.21%	0.00%
Case 4	Demand	1,274	15,095	212	525	1,293
	Adapted area	−349	262	35	52	0
		(−1.90%)	-1.42%	-0.19%	-0.28%	0.00%
Case 5	Demand	1,187	15,160	221	538	1,293
	Adapted area	−436	327	44	65	0
		(−2.37%)	-1.78%	-0.24%	-0.35%	0.00%
Case 6	Demand	1,187	15,698	221	0	1,293
	Adapted area	−436	865	44	−473	0
		(−2.37%)	-4.70%	-0.24%	(−2.57%)	0.00%
Case 7	Demand	1,187	15,919	0	0	1,293
	Adapted area	−436	1086	−177	−473	0
		(−2.37%)	-5.90%	(−0.96%)	(−2.57%)	0.00%

Under the current land-use policy (Case 0), the forested area will decrease and the built-up area will obviously increase. Specifically, the built-up area will increase from 1,187 ha in 1999 to 1,623 ha. in 2020. Meanwhile, the forested area will decrease from 15,160 ha (in 1999) to 14,833 ha (in 2020). Thus, in Case 0, the forested area will decrease by about 327 ha and the built-up area will increase by about 436 ha. 

### 2.4. Hydrological Model

In this study, the hydrological components were simulated by the Generalized Watershed Loading Functions (GWLF) model [22] based on the temperature and precipitation data derived from the six GCMs and land-use patterns discussed in the previous sub-sections. The GWLF model is a combined distributed/lumped parameter watershed model for simulating runoff, groundwater and streamflow. The daily water balance is calculated in an unsaturated zone and a shallow saturated zone. The water balance of an unsaturated zone is calculated as follows [23,24]:

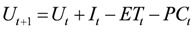
(2)
where *U_t+1_* (cm) and *U_t_* (cm) denote the moisture content of the root zone on days *t*+1 and *t*, respectively; *I_t_* denotes the infiltration I*_t_* (cm); *ET_t_* represents the evapotranspiration (cm) on day*t*; and *PC_t_* is the percolation (cm) into the deep saturated zone on day *t*. The infiltration is from that effect precipitation minus direct runoff and direct runoff is from SCS (Soil Conservation Service) curve number method, which generates direct runoff based on the precipitation, cover types, hydrological conditions, and hydrologic soil groups. Evapotranspiration is influenced by atmospheric conditions, land use and soil moisture content, which have the following relationship [24]:

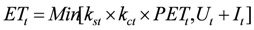
(3)
where *k_st_* and *k_ct_* are the coefficients of soil moisture stress and land cover respectively; and *PET_t_* is the potential evapotranspiration calculated by the Hamon equation, which is based on the number of daylight hours estimated under idealized conditions, and the saturated water vapor pressure [24,25]. The land cover coefficients of agricultural land, forested areas, built-up areas, grassland, and water bodies are 1, 1, 0.2, 0.83–1.25, and 1 respectively. Percolation occurs when the moisture in the soil of an unsaturated zone exceeds the field capacity. The water balance of a shallow saturated zone is calculated as follows:

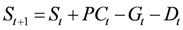
(4)
where S*_t_* (cm) denotes the water content of a shallow ground water aquifer at the beginning of day *t*; D*_t_* represents the deep seepage (cm) during day *t*; and G*_t_* (cm) is the amount of groundwater discharged into streams/rivers. The movement of water from the saturated zone to streams/rivers (G*_t_*) is regarded as a linear function of the moisture content of the saturated zone [23,24](. The equations describe the movement of water in a watershed. When the precipitation falls on the ground, the water is separated into runoff and infiltration. The infiltration and evapotranspiration will affect the water balance of unsaturated zone and the land use pattern will affect them at the same time. When the unsaturated zone is getting wet and saturated, the excessive water will move down to the saturated zone, which is the source of groundwater. Finally, the sum of groundwater and direct runoff becomes streamflow.

The GWLF model simulates the streamflow of the Wu-Tu watershed based on the historical streamflow data from 1995 to 2004. The parameters used in the model are the land cover coefficients, the evapotranspiration coefficients, the curve numbers of different land use types and the recession coefficient. All of these parameters are based on the condition of the watershed and not modified by calibration. Linear regression and the Nash-Sutcliffe efficiency [26] of the monthly observed streamflow and simulated streamflow are used to verify the hydrological parameters. In the model validation results, the Nash and Sutcliffe efficiency is 0.79 and R^2^ of the linear regression is 0.88. The regression model is significant at the 0.05 level. These results indicate that the hydrological model simulates the streamflow effectively.

### 2.5. Land-Use Allocation Model

We utilized the CLUE-s to allocate land for different uses based on the land-use demands in Cases 0–7 (Table 1). The CLUE-s (Conversion of Land Use and its effects at Small regional extent) was developed for the spatially explicit simulation of land use change. It is based on an empirical analysis of location suitability combined with the dynamic simulation of the competition and interaction between the spatial and temporal dynamics of land-use systems [27]. The relationships between land-use and its drivers can be fitted by using stepwise logistic regression. Furthermore, probability maps for all land-use types can be compiled by using logistic regression models. The explicit spatial allocation procedure converts non-spatial demands into land-use changes at various locations in the study area. The relationships between the types of land-use and their drivers are evaluated by the following stepwise logistic regression formula [27]:

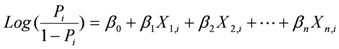
(5)
where *P_i_* denotes the probability of the occurrence of a particular land use type in a grid cell; *X_j,i_* are driving factors in grid cell *i* and driving factor *j*; and *β_j_* is the coefficient of each driving factor in the logistic model. After fitting the logistic regression models for all land use types, the restricted area and land use transition rules were specified for the study watershed. The restricted areas, which are environmentally sensitive to anthropogenic activities, were defined by the Construction and Planning Agency Ministry of the Interior, Taiwan. Finally, land-use changes were derived by the following iterative procedure based on the probability maps, the decision rules combined with the actual land-use maps and the demand for different types of land-use [27]. The first step of the iterative procedure makes a preliminary allocation by giving the iteration variable equal value for all land-use types. The procedure allocates the land use type with the highest total probability of occurrence in the considered grid cell [27]. The total area allocated for each type of land-use is then compared with the land-use demand. If the allocated area is smaller than the demanded area, the value of the iteration variable is increased. Conversely, if the allocated area is larger than the demanded area, the value of the iterative variable is reduced. The above steps are repeated until the land-use allocations satisfy the demanded areas in the current time step. The allocation procedure is then applied to the next yearly time step and so on until the target year is reached. Note that grid cells located in the restricted area cannot be converted to other land-use types during the allocation procedure.

## 3. Results

### 3.1. Climate Change Scenarios

Table 2 demonstrates the change rates in precipitation in 2020 compared with precipitation in 1999 in the GCMs. In the dry season (November–April), the estimated precipitation of all GCMs, except HADCM3, decreases by 7.04–15.51%; and in the wet season (May–October), the estimated precipitation declines slightly under GFDL21 and ECHAM5, but it increases by 6.34–16.25% under the other GCM models. The results indicate that climate change will cause precipitation to decrease in the dry season and increase in the wet season. Changes in annual precipitation will vary from −8.37% to +6.85% as the result of changes in precipitation during the two seasons. 

**Table 2:** The precipitation change in annual, in dry and wet seasons in 2020 in six GCMs.

	GCMs					
	GFDL21	ECHAM5	CGCM2	CCSM	INCM3	HADCM3
Annual	−5.16%	–8.37%	–2.95%	2.40%	6.85%	9.86%
Dry season	–8.75%	–14.28%	–15.51%	–7.04%	–8.16%	0.16%
Wet season	–2.19%	–3.48%	7.45%	6.34%	19.28%	16.25%

### 3.2. Hydrological Component Change on Land-Use Adaptation Policies

Before evaluating the adaptation of land-use patterns to the impact of climate change on hydrological components, we consider the effects of various climate change scenarios on hydrological components under the current land use policy (Case 0) and different land-use adaptation policies (Cases 1–7). In 1999, the annual groundwater, direct runoff, and streamflow in the study area were 1,676 mm, 2,216 mm and 3,802 mm, respectively. 

**Figure 2:**
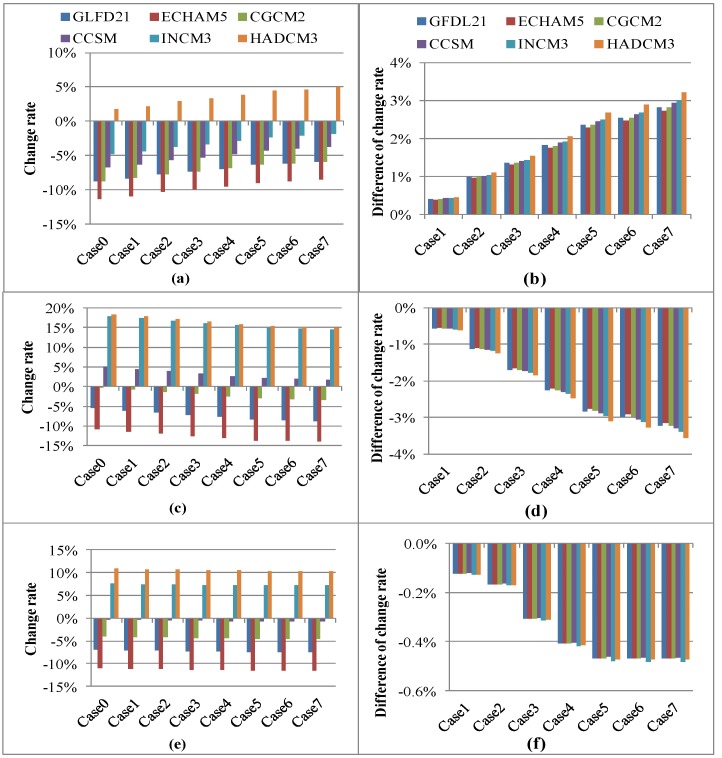
Annual hydrological components change and adaptive capacity with various GCMs and land use scenarios (**a**) & (**b**) Groundwater; (**c**) & (**d**) Direct runoff; (**e**) & (**f**) Streamflow.

Figure 2(a, c and e) compare the predicted annual groundwater, direct runoff, and streamflow rates under various climate change scenarios with the current rates for different land-use adaptation policies. Figure 2(b, d and f) show the difference of annual change rates of groundwater, direct runoff, and streamflow between various land-use adaptation cases and Case 0 (under the current land-use change policy). The change rate could be considered as the ability of land use adaptation. 

Figure 2(a) shows that, because of climate change, the annual groundwater rate will vary between −11.28% and 1.75% in Case 0, and between −8.98% and 4.43% in Case 5. Among the climate change scenarios, HADCM3 would result in the greatest increase in groundwater among the land-use adaptation policies. Specifically, under HADCM3, the groundwater rate will increase from 1.75% (Case 0) to 4.98% (Case 7). The differences in the annual groundwater rates are caused primarily by the decline in built-up land (2.37% of the total area in Cases 5, 6 and 7) and the increase in forested land (1.78%, 4.70% and 5.90% in Cases 5, 6 and 7 respectively), as shown in Table 1. Compared with the land-use scenario without adaptation (Case 0), groundwater will increase under all the land-use adaptation policies for the various climate change scenarios (Figure 2(b)). The most significant change occurs in Case 7, which has the largest change in the groundwater rate (3.22%) under the HADCM3 scenario, as shown in Figure 2(b). The results show that adapting land use patterns could reduce the impact of negative climate change on groundwater rates. 

Figure 2(c) shows the annual direct runoff in the current and future climate scenarios under different GCMs. These 6 GCMs predict either an increase or a decline in annual direct runoff. Among them, HADCM3 and INCM3 predict an increase from 14.88% (Case 7) to 18.43% (Case 0), and from 14.57% (Case 7) to 17.96% (Case 0), respectively. With an appropriate adaptation strategy, the negative impact of climate change on direct runoff could be reduced. Figure 2(d) demonstrates how land-use patterns can be adapted to changes in direct runoff. The land-use adaptation strategy in Case 7 reduces the direct runoff in Case 0 by 3.55% under the HADCM3 climate scenario. This is the largest reduction under the compared scenarios. 

The impacts of climate change on the annual streamflow under different land-use adaption strategies are shown in Figure 2(e). As the confluence effects on groundwater and direct runoff, the annual streamflow declines by 0.27–11.52% under GLFD21, ECHAM5, CGCM2 and CCSM, but increases by 7.15–10.86% under INCM3 and HADCM3. In Case 7, the adaptation strategy for the INCM3 climate scenario reduces the annual streamflow under the current land-use policy by 0.47% (Figure 2(f)). We found that the changes in the streamflow rates of Cases 5–7 are quite similar. This suggests that reducing the size of the built-up area would be the most effective way to adapt to climate change. 

**Figure 3:**
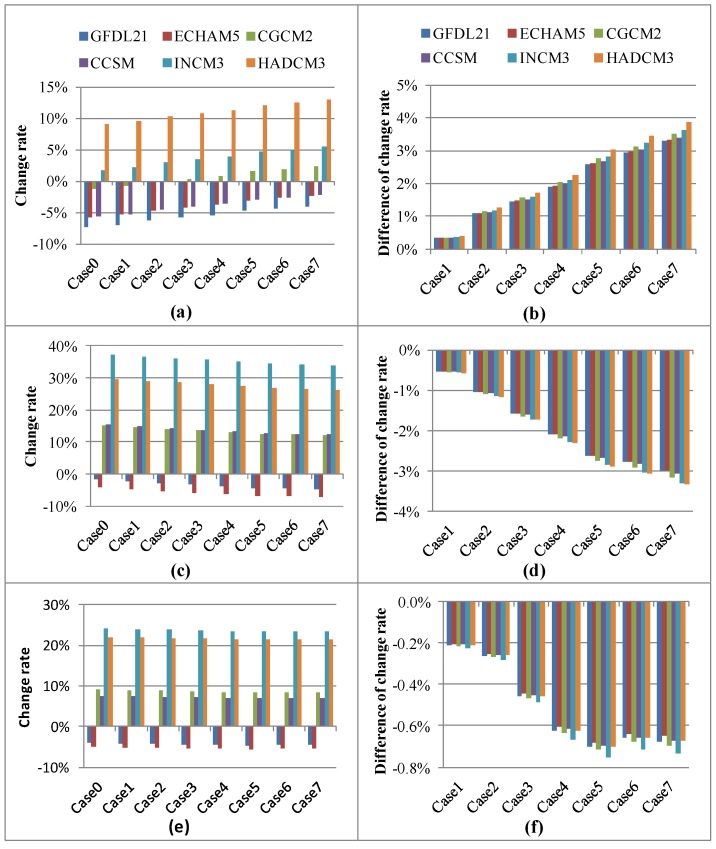
Hydrological components change and adaptive capacity with various GCMs and land use scenarios in wet season (**a**) & (**b**) Groundwater; (**c**) & (**d**) Direct runoff; (**e**) & (**f**) Streamflow.

**Figure 4:**
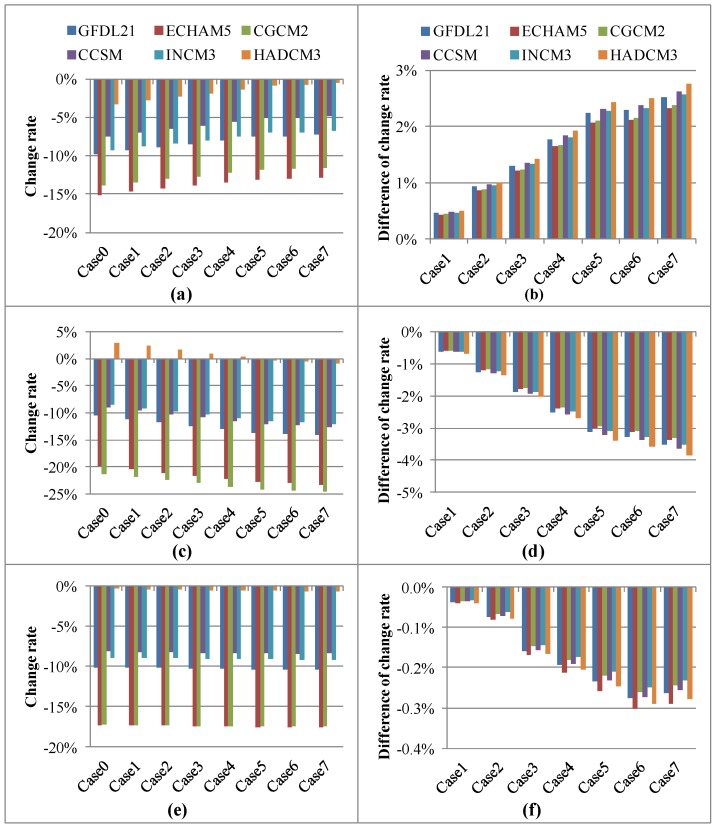
Hydrological components change and adaptive capacity with various GCMs and land use scenarios in dry season (**a**) & (**b**) Groundwater; (**c**) & (**d**) Direct runoff; (**e**) & (**f**) Streamflow.

Figure 3 (a, c and e) show the impacts of various GCMs on groundwater, direct runoff and streamflow in the wet season for different land-use adaptation strategies. Figure 3 (b, d and f) show the ability of land-use adaptations under different climate change scenarios on groundwater, direct runoff and streamflow in the wet season. Among the GCMs, HADCM3 yields the largest increase in groundwater, ranging from 9.06% (Case 0) to 12.94% (Case 7), compared to the groundwater rate without the impact of climate change (Figure 3(a)). Similar to the results for the annual groundwater rate (Figure 4(b)), all land-use adaptation strategies would increase the groundwater in the wet season. Case 7 yields the largest increase of 3.88% under HADCM3 climate scenario (Figure 3(b)). 

Figure 3(c) shows the impact of climate change on direct runoff in the wet season under various adaptation strategies. Generally, the change in direct runoff in the wet season is greater than that in annual direct runoff. Most of the GCMs result in greater direct runoff; and in some cases, the change of runoff in few GCMs is less than that under current climate conditions. For example, the change in direct runoff under the HADCM3 scenario ranges from 26.36% (Case 7) to 29.70% (Case 0). The decrease among land-use adaptation strategies indicates that adapting land-use patterns can minimize the impact of climate change on direct runoff. Therefore, a decrease of 3.34% in direct runoff can be expected in the wet season under the HADCM3 climate scenario and Case 7 land-use adaptation scenario (Figure 3(d)). 

Figure 3(e) shows the impact of climate change on streamflow in the wet season under various land-use adaptation strategies. The simulated precipitation has a direct impact on the direct runoff and streamflow. The increase in streamflow in the wet season under INCM3 is greater than that under HADCM3. This is because the simulated precipitation in the wet season under INCM3 is greater than that under HADCM3. According to the INCM3 GCM results, the streamflow rate in the wet season decreases from 24.15% in Case 0 to 23.42% in Case 7. Therefore, the adaptation strategy could make a 0.73% decrease of streamflow under the INCM3 climate scenario (Figure 3(f)). The results indicate that the adaptation strategy would increase the groundwater, but gradually reduce the direct runoff and streamflow in the wet season. 

Figure 4(a, c and e) show the impact of various climate change scenarios on groundwater, direct runoff and streamflow in the dry season for different land-use adaptation strategies. Figure 4(b, d and f) display the ability of land-use adaptation to the impact of climate change on the hydrological components in the dry season. In Figure 4(a), groundwater decreases in the dry season for all climate change scenarios because of reduced precipitation (Table 2). For the land-use patterns under the current land-use strategy (Case 0), the effect of climate change on the groundwater rate ranges from −15.10% to −3.27%. By considering the land-use adaptation strategy, the effect of climate change on groundwater can be reduced. For example, the impact of the HADCM3 climate change scenario on groundwater change is reduced to −0.84% when land-use adaptation strategy in Case 5 is adopted. The more that grassland and agricultural land are converted to forest, the smaller will be the impact of climate change on groundwater. Under the land-use adaptation scenarios in Cases 6 and 7, HADCM3 has the least impact on groundwater in the dry season with a decrease of −0.76% and −0.50%, respectively. In Figure 4(b), the largest increase in groundwater due to a land-use adaptation strategy is 2.77% under the HADCM3 climate scenario in Case 7. The maximum increase of groundwater in the dry season resulted by land-use adaptation strategies is smaller than those of annual groundwater and groundwater in wet season (Figure 2(b) and Figure 3(b)). This indicates that the land-use adaptation startegy is less effective in mitigating the effect of climate change in groundwater during dry season. 

As the results of reduction of precipitation in dry season (except HADCM3 scenario), direct runoff is reduced by 8.5%–24.54% for all land-use scenarios under the five GCMs scenarios (Figure 4(c)). HADCM3 is the only GCM scenario that shows an increase in precipitation during the dry season. Therefore, under current land-use patterns, direct runoff with an increase of 3.01% is expected. Furthermore, the effect of climate change on direct runoff can be reduced by 3.87% under Case 7, indicating the land-use adaptation strategy can be used to reduce the direct runoff change. Figure 4(d) demonstrates the ability of land-use adaptation to the climate change impact on direct runoff. For most GCM scenarios, the impact of land-use adaptation strategies on direct runoff is similar to the impact of climate change in the dry season. For the HADCM3 scenario, Case 7 has the greatest ability to adapt (3.84%) to the effect of climate change on direct runoff. Similar to the results of annual and wet-season direct runoff, the land-use adaptation strategy can reduce the impact of climate change on direct runoff in the dry season. Because of lower precipitation in the dry season, the streamflow is reduced by 0.37–17.60% for all GCM scenarios (Figure 4(e)). ECham5 and CGCM2 have the greatest impact on streamflow in the dry season with a reduction of 17.30–17.60% for various land-use adaptation cases. The small range of the effects of climate change for all land-use adaptation strategies indicates that the strategies have a limited impact in the dry season. Figure 4(f) shows the ability of land-use adaptations to the impact of climate change on streamflow in the dry season. A maximum reduction of 0.3% is expected in the streamflow during the dry season when Case 7 is adapted under the ECham5 climate scenario. Compared to the effect of land-use adaptation strategies on the annual and wet-season streamflow rates, the effect of the strategies in the dry season is not significant. The results also imply that the adaption strategies will increase the groundwater, but gradually reduce the direct runoff and streamflow in the dry season.

In this study, land-use development is controlled to mitigate the impact of climate change. The maximum capacity of mitigation from Case 0 to Case 7 is evaluated as adaptive capacity. The land-use adaptation strategies affect all the hydrological components under climate change listed in Table 3. In the cases of land-use development, the adaptive capacities of hydrological components are also different.

The adaptive capacities of groundwater are 3.22%, 3.88% and 2.77% in annual, wet and dry seasons, respectively. The adaptive capacities of groundwater stand only when adapting for decreasing groundwater. In addition, the capacities of direct runoff are 3.55%, 3.34% and 3.84% in annual, wet and dry seasons, respectively, while the runoff in future will increase. The capacities of streamflow are less than 1%. The management of land-use development and reducing the level of urbanization are two ways of adaptive capacity, but the ways of adaptation influence are various with different hydrological components.

**Table 3:** Adaptive capacity of climate change in the components by the land use management policy.

		Groundwater	Direct runoff	Stream flow
Annual	Increase	3.22%	X	X
	Decrease	X	3.55%	0.47%
Wet season	Increase	3.88%	X	X
	Decrease	X	3.34%	0.70%
Dry season	Increase	2.77%	X	X
	Decrease	X	3.84%	0.29%

Note: X means unavailable.

### 3.3. Land-Use Patterns Based on The Demands of Adaptations

**Figure 5:**
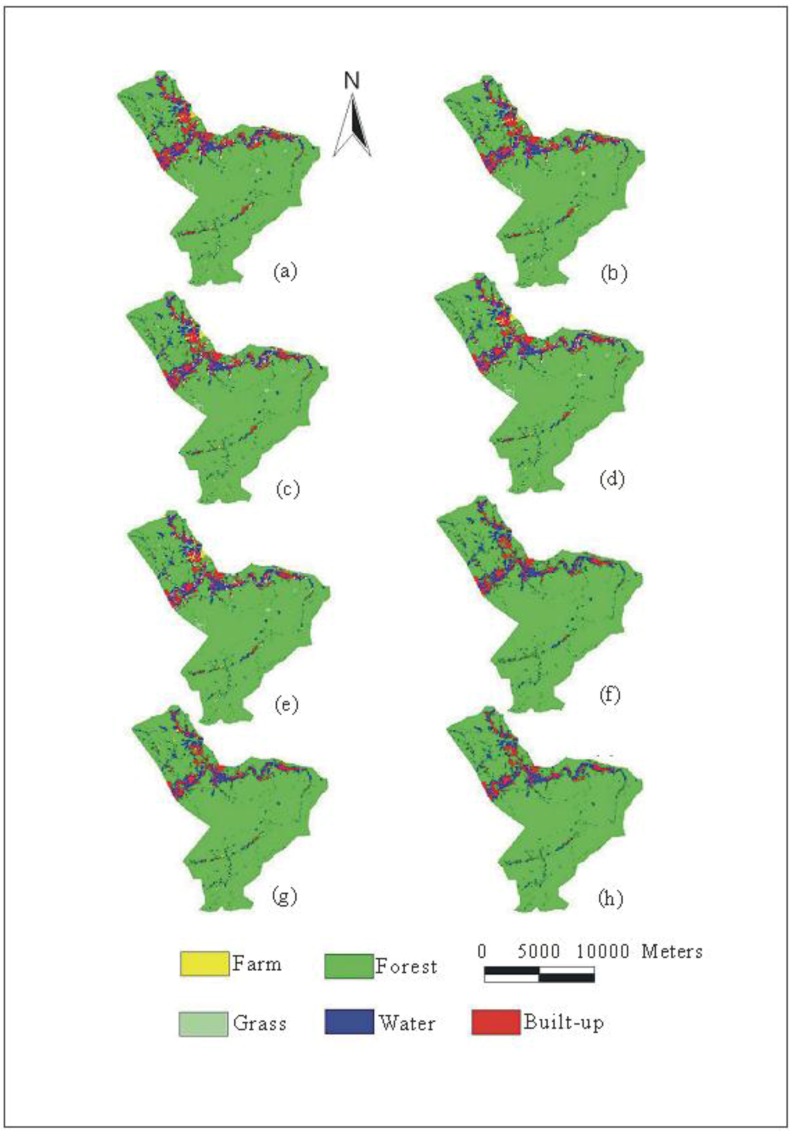
Spatial land-use distribution in the Wu-Tu watershed simulated by CLUE-s based on the different cases in 2020 (**a**–**h**: Case 0–Case7).

**Figure 6:**
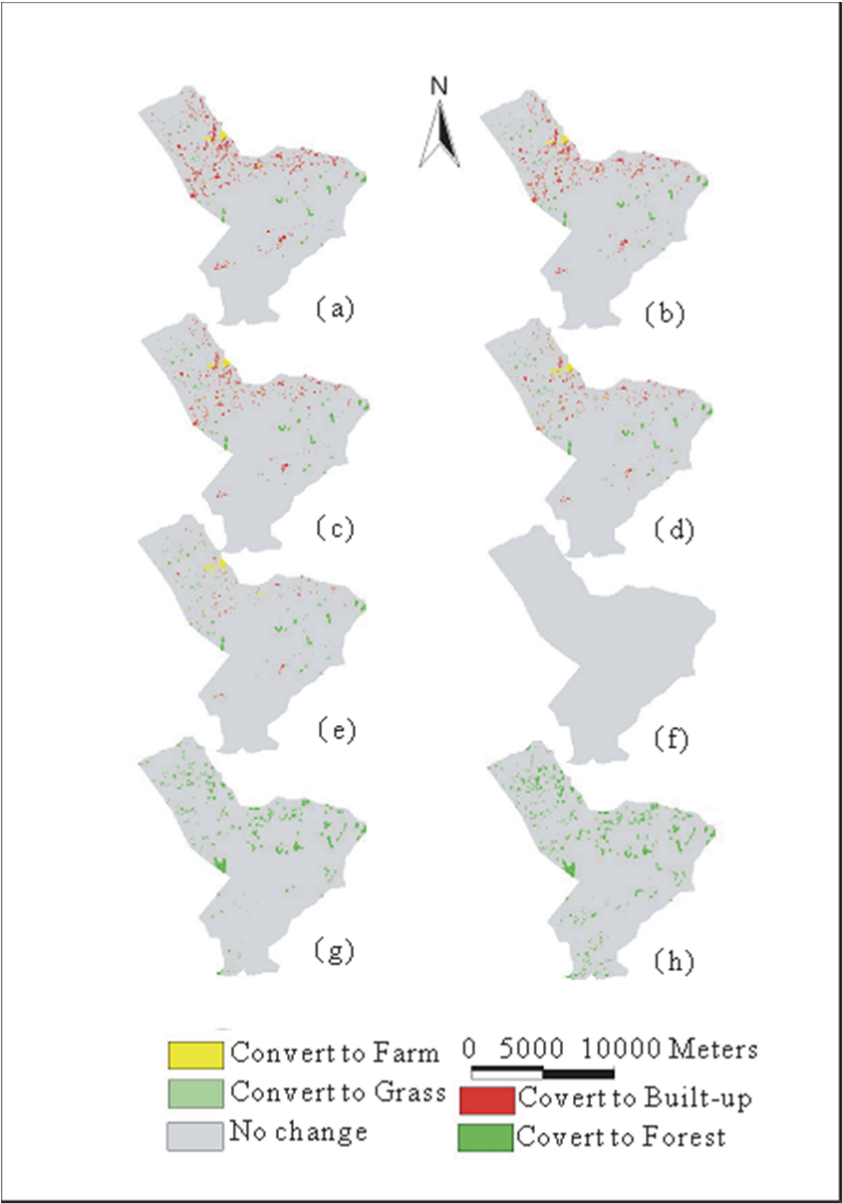
Land-use changes between 1999 and 2020 in (**a**) Case 0 (**b**) Case 1 (**c**) Case 2 (**d**) Case 3 (**e**) Case 4 and (**f**) Case 5 (**g**) Case 6 (**h**) Case 7.

Land-use patterns are predicted by the CLUE-s model based on various land-use demands (Cases 0–7). Figure 5 shows the predicted land-use patterns in 2020 under various policies using the CLUE-s model. The spatial land-use maps demonstrate that the land-use changes simulated by the model covers the entire study watershed, but the built-up parts are clustered in the middle and downstream areas. Generally, the area of built-up land increases in the study area, but areas of cultivated land, grassland and forest decrease. Figure 6 shows the patterns of land-use change between 1999 and 2020 for all cases. The results show that Case 0 is simulated based on the current policy that many regions are transformed to built-up lands spread over the downstream areas. Land-use change is varied gradually by the adaptation policies from Case 0 to Case 7, especially built-up land. As a result, the watershed gradually becomes less urbanized from Case 0 to Case 7, especially in the downstream area (Figure 6). 

## 4. Discussion

Proper management of LULC can create new opportunities by providing biophysical tools that increase the resilience of ecological systems and reduce the risk of catastrophic, irreversible events, such as local extirpation and global extinction [3]. For instance, the importance of incorporating climate change adaptation strategies into planning systems is now recognized in the UK, as shown by the planning policy guidelines [28] and guidelines for planning practitioners [29,30]. Moreover, adaptation was undertaken in a recent research project called “Adaptation Strategies for Climate Change in the Urban Environment (ASCCUE)” for strategic planning and urban design [31]. The goal of adaptation is defined as any adjustment that reduces the risk associated with climate change, or the vulnerability to climate change impacts, to a predetermined level without compromising economic, social, and environmental sustainability [32]. This study develops a framework for adopting land-use patterns based on land-use demands. The study looks at a watershed’s adaptability to climate change with the framework evaluated through the changes in hydrological components caused by land-use demands. 

Some studies have found that land use change is likely to affect water resources more significantly than climate change [33,34,35]. Other studies show that the impact of climate change on a region’s hydrology will be more significant than the impact of land-use changes [11,36,37,38,39,40,41]. These studies show that land-use patterns play a role in balancing the effects of climate change. In our study, the change in streamflow is 0.5% when only land-use change is considered (Case 7). Therefore, the impact of streamflow changes can be mitigated by efficient land management practices. Furthermore, our study suggests that there will be a wide range of changes in streamflow from −11% to 10%. The effects of the changes cannot be mitigated by land-management practices alone. 

In our land-use development scenarios, the maximum change is only 3.4% for the watershed. The adaptation scenarios of land-use change show a 7.45% increase in precipitation during the wet season, but there is no change in the groundwater. There is also a 0.16% increase in precipitation during the dry season, without any change in direct runoff. Of course, combining a policy of reduced urbanization and land management strategies may also yield interesting adaptation results and should be evaluated in the future. Naturally, watersheds exhibit different levels of adaptability. The relationship between economic variables and land-use scenarios is not considered in this study because our land-use predictions are based on population growth. If adaptation by changing land-use demand is practiced, it would certainly be necessary to examine the economic impacts and their implications. The study [42] assessed agricultural land-use patterns in terms of socio-economic development pathways. On a regional level, climate change patterns can accelerate land-use change in agricultural land-use models; hence, region specific values are needed for the models’ input parameters. 

This study does not consider the acceleration of land-use change due to climate-change patterns because urbanization is the major driver of the decline in agriculture land in the Wu-Tu watershed. Land use change associated with interactions of greenhouse gas emissions is important, and such information would be helpful building developing adaptive policies [17]. The immediate impact of climate change and the consequent adaptation of land-use patterns on a watershed scale should be evaluated before all the relevant factors are integrated. The results of this study also confirm that LULC-based strategies do not depend on remote political processes; that is, they can be used at local and regional levels to help achieve local conservation goals [3]. This empowers land managers to consider how their actions may contribute to land-use adaptation, or be may be counter-productive, under future conditions [3]. In addition, our results support the argument that the current global climate change agenda needs to recognize that climate change is a multidimensional issue, and that LULC must be included in global and regional strategies designed to mitigate the negative effects of climate change [16,17].

In the broadest terms, the success of an adaptation strategy or adaptation decision depends on how that action meets the objectives of adaptation, and how it affects the ability of others to meet their adaptation goals [8]. Moreover, proactive, rather than reactive, policy-making is essential in mitigating the effects of climate change and ensuring successful adaptation [17,43]. The foundation of strategic policy-making is anticipating and recognizing the risks and uncertainties associated with climate change and evaluating decision options under such uncertainty [17]). Given the high degree of uncertainty in the predictions of GCM’s, it is difficult to conclude that a particular land-use scenario would provide the best adaptation to climate change. Our study presents, practical land-use changes. In our results, annual precipitation under six GCMs shows different trends. Specifically, HADCM3, CCSM and INCM3 predict an increase in annual precipitation, while GFDL21, ECHAM5 and CGCM2 predict the opposite. Even so, five of the GCMs used in this study show some agreement about precipitation in the dry season. 

## 5. Conclusions

This work proposes a climate-change framework for land-use planning in a watershed. The framework’s predictions show that climate change will have a greater impact than land-use change on the hydrology of the studied watershed because of the small amount of land-use change. Although land-use change and climate change have different effects on hydrological components, the factors should not be considered in isolation when evaluating how the impact of climate change on a watershed’s hydrology affects land-use development. Reducing land-use development has a direct effect on the capacity of a watershed’s hydrology to adapt to climate change. The results of this study indicate that when climate change leads to increased precipitation, reducing land-use development results in a reduction in direct runoff and an increase in the base-flow. Moreover, reducing land-use development mitigates the impact of increased precipitation on direct runoff significantly during the dry season and on the base-flow in the wet season. Land-use allocations were made using the CLUE-s land-use model based on various land-use development scenarios. When considering climate and land-use change, direct runoff and base-flow should be major factors in determining local land-use demands designed to adapt to the impacts of climate change. The adaptability of land-use is marked by the amount of precipitation as well as by the hydrological components, but streamflow does not show a significant response to reductions in land-use development. Urbanization leads to an increase in direct runoff. Moreover, direct runoff decreases and the base-flow increases with a decline in land-use change. Thus, land-use adaptation on a local/regional scale provides an alternative way to reduce the impacts of global climate change on local hydrology.
